# Safety of third dose of COVID-19 vaccination in frail patients: Results from the prospective Italian VAX4FRAIL study

**DOI:** 10.3389/fonc.2022.1002168

**Published:** 2022-10-20

**Authors:** Serena Di Cosimo, Maria Teresa Lupo-Stanghellini, Massimo Costantini, Renato Mantegazza, Fabio Ciceri, Carlo Salvarani, Pier Luigi Zinzani, Alberto Mantovani, Gennaro Ciliberto, Antonio Uccelli, Fausto Baldanti, Giovanni Apolone, Sabina Delcuratolo, Aldo Morrone, Franco Locatelli, Chiara Agrati, Nicola Silvestris

**Affiliations:** ^1^ Department of Applied Research and Technological Development, Fondazione Istituti di Ricovero e Cura a Carattere Scientifico (IRCCS) Istituto Nazionale dei Tumori, Milano, Italy; ^2^ Hematology and Bone Marrow Transplantation Unit, Istituti di Ricovero e Cura a Carattere Scientifico (IRCCS) San Raffaele Scientific Institute, Milano, Italy; ^3^ Scientific Directorate, Fondazione Istituti di Ricovero e Cura a Carattere Scientifico (IRCCS) Istituto Nazionale dei Tumori di Milano, Milano, Italy; ^4^ Neuromuscular Diseases and Neuroimmunology Unit, Fondazione Istituti di Ricovero e Cura a Carattere Scientifico (IRCCS) Istituto Neurologico Carlo Besta, Milano, Italy; ^5^ University Vita-Salute San Raffaele, Milano, Italy; ^6^ Unità di Reumatologia, Azienda Unità Sanitaria Locale (USL)-Istituti di Ricovero e Cura a Carattere Scientifico (IRCCS), Reggio Emilia, Italy; ^7^ Unità di Reumatologia, Università degli Studi di Modena e Reggio Emilia, Modena, Italy; ^8^ Istituti di Ricovero e Cura a Carattere Scientifico (IRCCS) Azienda Ospedaliero-Universitaria di Bologna, Istituto di Ematologia “Seràgnoli”, Bologna, Italy; ^9^ Dipartimento di Medicina Specialistica, Diagnostica e Sperimentale, Università di Bologna, Bologna, Italy; ^10^ Humanitas Scientific Directorate, Istituti di Ricovero e Cura a Carattere Scientifico (IRCCS) Humanitas Research Hospital, Milan, Italy; ^11^ Department of Biomedical Sciences, Humanitas University, Milan, Italy; ^12^ William Harvey Research Institute, Queen Mary University, London, United Kingdom; ^13^ Scientific Directorate Istituti di Ricovero e Cura a Carattere Scientifico (IRCCS) Regina Elena, National Cancer Institute, Istituti Fisioterapici Ospitalieri (IFO), Rome, Italy; ^14^ Biotherapy Unit, Istituti di Ricovero e Cura a Carattere Scientifico (IRCCS) Ospedale Policlinico San Martino, Genoa, Italy; ^15^ Department of Neurosciences, Rehabilitation, Ophthalmology, Genetics, Maternal and Child Health, University of Genoa, Genoa, Italy; ^16^ Molecular Virology Unit, Fondazione Istituti di Ricovero e Cura a Carattere Scientifico (IRCCS) Policlinico San Matteo, Pavia, Italy; ^17^ Department of Clinical, Surgical, Diagnostics and Pediatric Sciences, University of Pavia, Pavia, Italy; ^18^ Scientific Directorate, Istituti di Ricovero e Cura a Carattere Scientifico (IRCCS) Istituto Tumori “Giovanni Paolo II”, Bari, Italy; ^19^ Scientific Directorate, San Gallicano Dermatological Institute Istituti di Ricovero e Cura a Carattere Scientifico (IRCCS), Rome, Italy; ^20^ Department of Pediatric Hematology and Oncology and Cell and Gene Therapy, Istituti di Ricovero e Cura a Carattere Scientifico (IRCCS) Ospedale Pediatrico Bambino Gesù, Roma, Italy; ^21^ Department of Gynecology-Obstetrics and Pediatrics, University “La Sapienza”, Roma, Italy; ^22^ Cellular Immunology Laboratory, National Institute for Infectious Diseases L Spallanzani – Istituti di Ricovero e Cura a Carattere Scientifico (IRCCS), Rome, Italy; ^23^ Medical Oncology Unit, Department of Human Pathology “G. Barresi”, University of Messina, Messina, Italy

**Keywords:** COVID – 19, vaccination, frail adults, adverse (side) effects, safety

## Abstract

**Importance:**

Despite people with impaired immune competence due to an underlying disease or ongoing therapy, hereinafter frail patients, are (likely to be) the first to be vaccinated, they were usually excluded from clinical trials.

**Objective:**

To report adverse reactions of frail patients after receipt of the third dose (booster) administered after completion of a two-dose mRNA vaccination and to compare with those reported after the receipt of the first two doses.

**Design:**

A multicenter, observational, prospective study aimed at evaluating both the safety profile and the immune response of Pfizer-BioNTech or Moderna vaccines in frail patients.

**Setting:**

National Project on Vaccines, COVID-19 and Frail Patients (VAX4FRAIL)

**Participants:**

People consenting and included in the VAX4FRAIL trial.

**Exposure:**

A series of three doses of COVID-19 mRNA vaccination from the same manufacturer.

**Main outcome(s) and measure(s):**

Evaluation of a self-assessment questionnaire addressing a predefined list of eight symptoms on a five-item Likert scale. Symptoms were classified as severe if the patient rated them as severe or overwhelming.

**Results:**

Among 320 VAX4FRAIL participants diagnosed/treated for hematological malignancies (N=105; 32.8%), solid tumors (N=48; 15.0%), immune-rheumatological diseases (N=60; 18.8%), neurological diseases (N=107; 33.4%), and receiving the booster dose, 70.3% reported at least one loco-regional or systemic reactions. Adverse events were mostly mild or moderate, none being life-threatening. Only six of the 320 (1.9%) patients had their treatment postponed due to the vaccine. The safety profile of the booster compared to previously administered two doses showed a stable prevalence of patients with one or more adverse events (73.5%, 79.7% and 73.9% respectively), and a slightly increment of patients with one or more severe adverse events (13.4%, 13.9% and 19.2% respectively).

**Conclusions and relevance:**

The booster of the mRNA COVID-19 vaccine was safely administered in the largest prospective cohort of frail patients reported so far. VAX4FRAIL will continue to monitor the safety of additional vaccine doses, especially systemic adverse events that can be easily prevented to avoid interruption of continuity of care.

**Clinical trial registration:**

https://clinicaltrials.gov/ct2/show/NCT04848493, identifier NCT04848493.

## Introduction

In the autumn of 2021, health authorities worldwide recommended an additional dose of approved messenger RNA (mRNA) vaccines against COVID-19 after completing a series of primary vaccinations (1, 2). This implementation of the vaccine campaign was based on the results of a phase III clinical trial conducted by Pfizer-BioNTech to overcome the waning immunity of primary vaccination and protect against delta and upcoming variants (3).

Although the major impact of SARS-CoV-2 infection on people with frailties justify an approach in which vaccination is offered first, they were not included in the official clinical trial. Frail patients, i.e., subjects with impaired immune competence due to their underlying diseases or ongoing therapies may have been excluded because of the concern that a vaccine trial could compromise the metabolism of their treatment, causing worsening of disease specific symptoms. Moreover, frail people may suffer different side effects to the vaccine compared with healthy adults. Of note, while the term and concept of frailty technically most often refers to a multidimensional process and state of age-related physiological decline, we used the term “frail” for high-risk immunocompromised patients affected by active hematological, solid malignancies, immune-rheumatological disease, and neurological disease. Herein, we report the safety results of the third dose of mRNA SARS-CoV-2 vaccination within the Italian, multicenter, prospective, observational VAX4FRAIL study, designed specifically to monitor response to COVID-19 vaccines in frail patients (4, 5).

## Patients and methods

Coinciding with the authorization of an additional dose for people with immune-compromised conditions (4), the VAX4FRAIL study collected participants’ information on adverse reactions to extra doses of the COVID-19 vaccine. Patients aged 18 years or more, who had received COVID-19 vaccination with mRNA vaccines (BNT-162b2 Pfizer-BioNTech or m-RNA-1237 Moderna), and completed the assessment questionnaire after the third dose were considered eligible for this analysis. The eligible population included frail patients with a diagnosis of active hematological, solid malignancies, immune-rheumatological disease, and neurological disease. Note that malignancy was considered as active in the presence of tumor disease and/or systemic anti-cancer treatment; and immune suppression was needed to qualify for the rheumatologic or neuromuscular cohort. Details on the eligibility criteria of the four VAX4FRAIL study groups are reported in **Table 1**. Demographic data, local and systemic self-reported and self-evaluated reactions, and health impacts during the first week after booster vaccine administration were collected. A questionnaire assessing how much the patient was troubled by a predefined list of eight symptoms on a five-item Likert scale (not at all, slightly, moderately, severely, overwhelming) was administered to each one. (**Table S1** in **Supplementary Material**). For the purpose of this study a symptom was considered present when the patient choice was slightly or moderately, and severe when it was severely or overwhelming.

**Table 1:** Eligibility criteria of the four main groups of patients enrolled in VAX4FRAIL study.

HEMATOLOGICAL MALIGNANCIES	SOLID TUMORS	IMMUNORHEUMATOLOGICAL DISEASES	NEUROLOGICAL DISEASES
1a. Newly diagnosed patients with ANY hematological malignancy requiring treatment. 1b. Patients with ongoing treatments or with treatments completed within 6 months (chemotherapy and target therapies) other than antibodies. More specifically: patients with ongoing or completed chemotherapy or patients with ongoing or completed Ibrutinib or patients with ongoing or completed ruxolitinib 1c. Patients treated with anti-CD19 or CD20 or CD22 or CD30 or anti-PD1 antibodies with or without chemotherapy OR patients receiving CAR-T cells: patients treated anti-B-cell or patients treated anti-CD30 or patients treated anti-PD1. 1d. Patients at three months after autologous or allogeneic transplantation without active immune suppressive therapy: after autologous transplantation or after allogenic transplantation.	2a. Chemotherapy in adjuvant therapy. All patients with a diagnosis of solid tumors apart resected basal-cell or squamous-cell carcinoma of the skin, melanoma in situ, carcinoma *in situ* of the cervix, and carcinoma *in situ* of the Breast. Under curative surgery (stage II-III) for the solid tumor or hemotherapy alone or in combination with target therapies or radiotherapy. 2b. Chemotherapy in metastatic Disease. All patients with a diagnosis of solid tumors with Metastatic disease (stage IV), undergoing chemotherapy alone or in combination with immunotherapy or target therapy. 2c. Immunotherapy in metastatic Disease. All patients with a diagnosis of solid tumors with Metastatic disease (stage IV), undergoing immunotherapy alone. 2d. Target therapies in metastatic Disease. All patients with a diagnosis of solid tumors with Metastatic disease (stage IV), Undergoing target therapy alone.	3a. Patients with ANCA-associated vasculitis classified according to Chapel Hill Consensus Conference nomenclature, treated with immunodepressants agents with/without glucocorticoids or treated with RTX with/without glucocorticoids. 3b. Interstitial Lung Disease in Autoimmune Conditions. Patients with a diagnosis of a specific CTD, myositis or rheumatoid arthritis based on validated classification criteria, and clinically significant ILD defined as disease treated with traditional immunodepressants or rituximab and fibrotic and/or inflammatory changes on chest CT not attributable to infection, and no evidence of obstructive lung disease. Patients treated with traditional immunodepressive agents with/without glucocorticoids or patients treated with rituximab with/without glucocorticoids.	4a. Patients with a diagnosis of multiple sclerosis, age < 60 years with relapsing-remitting MS on Ocrelizumab (anti-CD20 monoclonal antibody) or with secondary/primary progressive MS on Ocrelizumab (anti-CD20 monoclonal antibody). 4b. Generalized Myasthenia Gravis, on immunosuppressive polytherapies or on B-cell targeted biological treatments, with lymphocytes count < 1 cell/microliter, or with thymoma.

Methodology of administration of the questionnaire and collection and grading of adverse events were previously described (6). The questionnaire was in paper-and-pen format and it was administered by nursing-staff during the follow-up visit, after consent from the patients. The prevalence of symptoms after the booster dose was compared with that observed among patients who received the first and the second dose of mRNA vaccine. For this analysis, we used the sample of patients who filled in the questionnaire after the three administrations of vaccines. The protocol has been approved by national competent authorities (AIFA) and the ethics committee of the National Institute for Infectious Diseases Lazzaro Spallanzani (IRCCS).

## Results

Between September 2021 and March 2022, 401 VAX4FRAIL eligible patients received a third dose of COVID-19 vaccination. Three-hundred and twenty (79.8%) patients filled-in the questionnaire. There were no significant differences between questionnaire responders and non-responders according to age, (P=0.322), sex (P=0.124) and type of vaccine (P=0.527) (**Table 2**). Conversely, a significant higher compliance was observed among BNT162b2 (83.3%) as compared to mRNA-1273 vaccine (70.1%) recipients (P=0.003), and for patients with neurological diseases (91.5%) as compared to patients with solid tumors (62.3%) or immune-rheumatological diseases (68.9%) (P<0.001) (**Table 2**).

**Table 2:** Characteristics of overall VAX4FRAIL eligible patients (N= 401) and self-assessment questionnaire responders (N= 320).

	Eligible No.		Self-assessment No. (%)	Test for heterogeneity
Age (years)				
18-50	107		86 (80.4)	
51-70	218		169 (77.5)	
> 70	76		65 (85.5)	P-value = 0.322
Sex				
Males	207		159 (76.8)	
Females	194		161 (83.0	P-value = 0.124
Main diagnostic groups				
Hematological malignancies	131		105 (80.2)	
Solid tumors	77		48 (62.3)	
Immunorheumatological diseases	76		60 (68.9)	
Neurological diseases	117		107 (91.5)	P-value < 0.001
Vaccine				
mRNA-1237 Moderna	107		75 (70.1)	
BNT-162b2 Pfizer BioNTech	294		245 (83.3)	P-value = 0.003
Total	401		320 (79.8)	

The 320 patients of the study population were affected by hematological malignancies (N=105; 32.8%), solid tumors (N=48; 15.0%), immune-rheumatological diseases (N=60; 18.8%), neurological diseases (N=107; 33.4%). Seventy-five (23.4%) received mRNA-1273 vaccine, and 245 (76.6%) received BNT162b2.; 159 (49.7%) were females, their median (range) age being 59 years (18 to 91). The median (range) interval from completion of the primary COVID-19 vaccination series to receiving the booster was 168 days (82 to 302).

Overall, after the booster dose, 225 (70.3%) reported at least one local or systemic reaction occurred in the first week after the third dose (55, 17.3% were severe). The most common solicited local adverse event was pain at the injection site (59.7%, 7.2% were severe), while fatigue (35.3%, 4.7% were severe), bone pain (29.4%, 5.6% were severe) and fever (16.6%, 5.6% were severe) were the most common solicited systemic adverse events (**Table 3**). Overall, 6 patients (1.9%) after the booster dose required postponement of specific treatment for underlying medical conditions (four with a immune-rheumatological disease, one with a neurological disease and one with a solid tumor). No serious adverse events required hospitalization related to the vaccine were recorded.

**Table 3:** Vaccine-related reactions over the week after the third (booster) dose.

	Not at all N (%)	Mild N (%)	Moderate N (%)	Severe N (%)	Overwhelming N (%)	Mild to Overwhelming N (%)	Severe to Overwhelming N (%)
Pain at the injection site	129 (40.3)	119 (37.2)	49 (15.3)	19 (5.9)	4 (1.3)	191 (59.7)	23 (7.2)
Fatigue	207 (64.7)	64 (20.0)	34 (10.6)	12 (3.8)	3 (0.9)	113 (35.3)	15 (4.7)
Headache	260 (81.3)	35 810.9)	18 (5.6)	6 (1.9)	1 (0.3)	60 (18.8)	7 (2.2)
Bone pain	226 (70.6)	53 (16.6)	23 (7.2)	15 (4.7)	3 (0.9)	94 (29.4)	18 (5.6)
Fever	267 (83.4)	30 (9.4)	5 (1.6)	16 (5.0)	2 (0.6)	53 (16.6)	18 (5.6)
Enlarged lymph nodes	311 (97.2)	4 (1.3)	4 (1.3)	–	1 (0.3)	9 (2.8)	1 (0.3)
Skin rash	315 (98.4)	3 (0.9)	1 (0.3)	–	1 (0.3)	5 (1.6)	1 (0.3)
Insomnia	296 (92.5)	17 (5.3)	4 (1.3)	2 (0.6)	1 (0.3)	24 (7.5)	3 (0.9)
Diarrhoea	305 (95.3)	9 (2.8)	5 (1.6)	–	1 (0.3)	15 (4.7)	1 (0.3)
Nausea or vomiting	304 (95.0)	9 (2.8)	5 (1.6)	–	2 (0.6)	16 (5.0)	2 (0.6)

**Figure 1:**
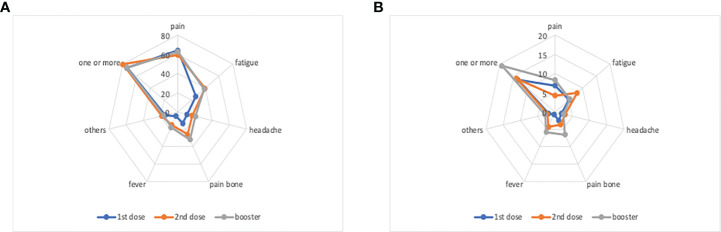
Prevalence of solicited adverse events within 7 days following third-dose vaccination. **(A)** Any-grade local and systemic adverse events. **(B)** Severe local and systemic adverse events.

The analysis of adverse reactions after the first, the second and the booster dose (on 276 patients with the three questionnaires filled in), showed a stable prevalence of patients with one or more adverse events (73.5%, 79.7% and 73.9% respectively), and a slight increment of patients with one or more severe adverse events (13.4%, 13.9% and 19.2% respectively) - **Figure 1**. This increment was mainly due to more patients suffering severe pain after the booster dose (6.9%, 4.3% and 8.3% respectively), bone pain (2.5%, 3.6% and 6.5% respectively), and fever (0.7%, 4.3% and 5.8% respectively).

## Discussion

Our data confirm that COVID-19 vaccination is safe in the largest prospectively reported series of patients with impaired function of the immune system reported to date. No unexpected adverse reactions occurred in frail patients receiving an additional dose of the COVID-19 vaccine. These findings are consistent with previous reports on the general population. Specifically, the Pfizer-BioNTech clinical trial, including 306 persons aged 18-55 years, showed that reactions after the third dose were comparable to those reported after the second dose (3). These adverse reactions included mild to moderate systemic and injection site reactions (3). Similarly, the patterns of adverse reactions observed after the third dose of the Moderna vaccine were consistent with previously described responses after receiving the second dose (7). Since the initial trials, the safety of the vaccines has continued to be evaluated and in the general population reactions to subsequent doses, even to the fourth, are similar to those previously reported and self-limiting (8).

Scattered evidence shows that adverse events are uncommonly reported in frail people and they are generally of mild or moderate severity (9–11). Specifically, up to 50% of vaccinated cancer patients experience no toxicity after the first dose of vaccine (as compared to 30% or less of healthy controls). Moreover, adverse reactions are not increased by the second dose (10), except in cases reporting toxicity from the beginning of the vaccination program (6). Intriguingly, we found that severe reactions were slightly more common after the third vaccination dose. Correlating toxicity with vaccine response is rather tricky, but we cannot ignore that a livelier immune response is associated with more side effects. For example, in the initial efficacy and safety study of mRNA vaccines (12, 13), younger recipients aged between 16 and 55 years reported systemic events more frequently than their older ≥55 years counterparts (6, 11). In addition, increased side effects were reported after the second than after the first dose, consisting mainly of systemic symptoms such as fatigue and headache. Therefore, the booster toxicity profile observed in VAX4FRAIL could simply reflect the capability of the third dose of eventually eliciting a specific immune response also in the fragile patient population, as we recently reported (14). All the reported toxicities we observed are still clinically manageable and have a negligible effect on the cancer care path as already reported (6, 15). VAX4FRAIL will continue to monitor vaccine safety, including additional doses of COVID-19 vaccine, and provide data to guide vaccine recommendations and protect public health.

## Data availability statement

The raw data supporting the conclusions of this article will be made available by the authors, without undue reservation.

## Ethics statement

The studies involving human participants were reviewed and approved by national competent authorities (AIFA) and the ethics committee of the National Institute for Infectious Diseases Lazzaro Spallanzani (IRCCS). The patients/participants provided their written informed consent to participate in this study.

## Author contributions

MC had full access to all the data in the study and took responsibility for the integrity of the data and the accuracy of the data analysis. Concept and design, SDC, MTLS, MC, NS, GA, and AMa. Acquisition, analysis, or interpretation of data, SDC, MTLS, MC, and NS. Drafting of the manuscript, SDC, MTLS, MC, and NS. Critical revision of the manuscript for important intellectual content, all authors. Statistical analysis, MC. All authors contributed to the article and approved the submitted version.

## Funding

This study was financed by the Italian Ministry of Health within Ricerca Corrente 2021-Special Projects-VAX4FRAIL.

## Acknowledgments

We are indebted for their precious support to this study the Research Director Dr. Giuseppe Ippolito, and the Deputy General Director for Health Research and Innovation, Dr. Gaetano Guglielmi, of the Italian Ministry of Health. We would also like to thank the patients who were enrolled in the VAX4FRAIL study and their families, the research nurses, the administrative staff and all those who will be actively involved in their continuous care, study data collection and analysis, and ultimately in the scientific production that will result from this research. A heartfelt acknowledgment to the whole VAX4FRAIL study group.

## Conflict of interest

SDC reports payments as speaker bureau Pierre-Fabre, AstraZeneca, as hoc advisor MEDSIR, IQVIA. AMa reports royalties for reagents related to innate immunity, consulting fees and payment or honoraria as a consultant/advisory board member for Novartis, Roche, Ventana, Pierre Fabre, Verily, AbbVie, BMS, J&J, Imcheck, Myeloid Theraputics, Astra Zeneca, Biovelocita, BG Fund, Third Rock Venture, Violend Verseau Therapeutics, Macrophage pharma, Ellipses Pharma, and Olatec Therapeutics, and is the inventor of patents related to PTX3 and other innate immunity molecules. RM reports consulting fees paid to 2 author from Alexion, Argenx, and UCB, payment to author for lectures, presentations, speakers 3 bureaus, manuscript writing or educational events from Alexion, Argenx, Merck Serono, Reflexion 4 Medical Network, Sanofi Aventis, UCB, paid participation on Data Safety Monitoring or Advisory Board with Alexion, Argenx, Catalyst, and UCB. AU reports grants or contracts unrelated to this work from FISM, ALEXION, BIOGEN, ROCHE, MERCK SERONO, and COVAXIMS, participation on Data Safety Monitoring or Advisory Board for BD, BIOGEN, IQVIA, SANOFI, ROCHE, ALEXION, BRISTOL MYERS SQUIBB. NS reports payment or honoraria for lectures, presentations, speakers bureaus, manuscript writing or educational events from Lilly, Roche, and Servier.

The remaining authors declare that the research was conducted in the absence of any commercial or financial relationships that could be construed as a potential conflict of interest.

## Publisher’s note

All claims expressed in this article are solely those of the authors and do not necessarily represent those of their affiliated organizations, or those of the publisher, the editors and the reviewers. Any product that may be evaluated in this article, or claim that may be made by its manufacturer, is not guaranteed or endorsed by the publisher.
